# How not to waste a crisis: a qualitative study of problem definition and its consequences in three hospitals

**DOI:** 10.1177/1355819619828403

**Published:** 2019-03-01

**Authors:** Graham Martin, Piotr Ozieranski, Myles Leslie, Mary Dixon-Woods

**Affiliations:** 1 Director of Research, THIS Institute (The Healthcare Improvement Studies Institute), University of Cambridge, UK; 2 Lecturer, Department of Social and Policy Sciences, University of Bath, UK; 3 Assistant Professor, Department of Community Health Sciences, University of Calgary, Canada; 4 Health Foundation Professor of Healthcare Improvement Studies, THIS Institute (The Healthcare Improvement Studies Institute), University of Cambridge, UK

**Keywords:** hospitals, patient safety, qualitative, quality, social problems

## Abstract

**Objectives:**

The prominence given to issues of patient safety in health care organizations varies, but little is known about how or why this variation occurs. We sought to compare and contrast how three English hospitals came to identify, prioritize and address patient safety issues, drawing on insights from the sociological and political science literature on the process of problem definition.

**Methods:**

In-depth qualitative fieldwork, involving 99 interviews, 246 hours of ethnographic observation, and document collection, was carried out in three case-study hospitals as part of a wider mixed-methods study. Data analysis was based on the constant comparative method.

**Results:**

How problems of patient safety came to be recognized, conceptualized, prioritized and matched to solutions varied across the three hospitals. In each organization, it took certain ‘triggers’ to *problematize* safety, with crises having a particularly important role. How problems were constructed – and whose definitions were prioritized in the process – was highly consequential for organizational response, influencing which solutions were seen as most appropriate, and allocation of responsibility for implementing them.

**Conclusions:**

A process of problem definition is crucial to raising the profile of patient safety and to rendering problems amenable to intervention. How problems of patient safety are defined and constructed is highly consequential, influencing selection of solutions and their likely sustainability.

## Introduction

Recent years have seen huge public interest, policy attention and organizational endeavours focused on the quality and safety of health care. The reasons for this are multiple, including a series of scandals and high-profile incidents,^1^ a growing mass of evidence on the epidemiology of avoidable harm,^2–4^ and the emergence of the modern patient safety movement and associated organized advocacy.^5^ Perhaps less remarked has been the variability in attention given to different topics in quality and safety. For example, while surgery has seen welcome improvements over time, the safety of those with mental illness has received rather less attention in research and policy.^6^ Yet questions of how problems of quality and safety are defined and identified, what counts as a solution, and how those choices influence practice have received scant attention in the literature.

In this paper, we offer an analysis of how problems of patient safety come to be recognized, conceptualized, prioritized and matched to solutions in health care organizations. We use three qualitative case studies of English National Health Service (NHS) hospital trusts as our data sources. Our analysis draws on an important social science tradition that describes the genesis and trajectory of ‘social problems’.^7–10^ These accounts emphasize that social problems cannot be treated as self-evident facts: instead, problems are constructed or created by various collective social processes that themselves are dynamic and contingent.^11–13^ As a result, issues may be defined as problems at some times but not others, and their prominence may amplify or diminish over time, for example at the level of health policy formation,^14–16^ or in the work of social movements to orchestrate action around quality and safety.^17,18^

Recent research has begun to examine different problematizations in health care, showing, for example, how dominant constructions, articulated through standards and measures, may be misaligned with ‘local’ understandings of what needs to be improved and how.^19,20^ However, examination of the processes by which dominant groups, such as senior managers in health care organizations, construct and advance particular problematizations of patient safety issues has been limited. As Cornelissen and Werner note, such top-down constructions of problems can be taken for granted,^21^ yet they too rely on active work ‘to mobilize potential adherents and constituents, to garner bystander support, and to demobilize antagonists’.^22^

Here, we seek to apply the insights of the problem-definition literature to understand how those in senior managerial positions in health care organizations (sometimes known as the ‘blunt end’, as opposed to the ‘sharp end’ of care, where patient-facing clinical activities take place^23^) come to identify and characterize patient safety problems and organize responses. Across the three case studies that form our data, our focus is on the social processes of *problematization* – how issues come to be identified and understood as problems that required action – and the consequences of that problematization for their resonance with staff and for allocation of responsibility.

## Methods

In-depth qualitative fieldwork, involving interviews, ethnographic observation and document collection, was carried out in three purposively chosen hospital trusts, as part of a wider mixed-methods study.^24–26^ At the time of study, each site was undertaking concerted, hospital-wide efforts to improve quality and safety, which formed a key focus of our data collection. All three were teaching hospitals with close relationships with nearby medical schools; two (‘Appleby’ and ‘Berryton’) were acute trusts providing a wide range of secondary care services to large local catchments; the third (‘Cherryville’) was a tertiary centre providing specialist services.

The balance of qualitative data-collection techniques varied by site, but in all three sought to cover both the ‘blunt end’ (senior clinicians and managers responsible for devising and implementing initiatives) and ‘sharp end’ (clinicians responsible for delivering care, and the subject of efforts to improve culture, processes and behaviour). Having obtained ethical and research governance clearances, we undertook fieldwork that included: (i) qualitative interviews with blunt- and sharp-end participants on safety in general and the particular approach adopted in their hospital; (ii) ethnographic observation of day-to-day care processes in a selection of units in Appleby (acute wards and maternity services) and Berryton (acute wards). We conducted 64 interviews and 56 hours’ observation in Appleby, 11 interviews and 190 hours’ observation in Berryton, and 24 interviews in Cherryville (where observations were not possible). As we undertook the main analysis of our data, the implications of constructions of quality and safety at the blunt end became an increasingly interesting focus of inquiry, and we therefore undertook further analysis to deepen our understanding of how it was received and acted upon at the sharp end.

In undertaking this additional analysis, we deployed an approach based on the constant-comparative method,^27^ assisted by NVivo software. Our ‘sensitizing concepts’ came from the social scientific literature on problem definition. Initially, we coded data excerpts that related broadly to these themes, before developing a coding framework that distinguished different parts of the process of defining problems and identifying appropriate solutions and facilitated cross-case comparison.

## Results

We found that patient safety was acknowledged as a priority both by senior leaders of the hospitals and by clinicians delivering care, but that it was in competition with other organizational priorities for the inherently limited space on the organizational agenda.^28^ We examine in turn how each organization sought to reassert the primacy of safety concerns (see also Table 1). We found in particular that in each organization it took certain ‘triggers’ to *problematize* safety – bring it into being as a social problem – such that it was positioned at the forefront of organizational concerns and seen as something amenable to action. The form taken by these triggers, however, differed across cases. How problems were constructed – and whose definitions were prioritized in the process – then had important consequences for the organization’s response.

**Table 1.** The construction of problems, resolutions and responsibilities in the three sites.

	Appleby	Berryton	Cherryville
Problem as constructed by ‘blunt end’	*Behavioural: an issue of knowledge of appropriate standards of behaviour, and sustaining behavioural change through time*‘A number of action plan come back, and they are not fully implemented. So they are revisited, and the message is reinforced, and if we have to we will have to reaudit them again just to make sure. But the whole idea is about getting lessons learnt actually adopted, […] So it’s just a matter of education, re-education’. (Non-executive director)	*Behavioural and system-related: an issue of standards of behaviour, and of systems acted as latent causes of error*‘It’s as much the organization’s fault as the individual. There will be some individuals – you’ll have the one, two per cent who don’t care basically, who have just lost the plot completely – and we need to weed those out. But the vast majority of people want to do a good job’. (Chief operating officer)	*Cultural: an issue of making patient safety a central and routine concern for all members of staff*‘We wanted to make breaches of patient safety and quality stigmatised. We wanted to make it clear that no, it’s not acceptable to give most of the tablets, but not all of them. That’s not really on. And so we had lots of posters all around the hospital. Every ward had these little charts on the wall, where they had to mark on it, when was their last C diff case, when was their last missed drug, all that sort of stuff’. (Head of Surgery)
Approach to resolution	*Education and enforcement of behavioural standards*‘We drafted the policy, it made sure everyone knew what they should be doing, so that they knew what our expectations of them were. […] I think that is one aspect that is missing from the health service. I am not saying there should be a discipline code and things of that nature, I am not saying that at all, but I do find the culture in the health service, is very, sort of, aspirational: Let’s lead them and they will learn and follow, when in actual fact sometimes people need to know that there is a consequence for your actions, or more importantly for your inactions’. (Non-executive director)	*Socio-technical: better design of systems to reduce latent causes; enforcement of behavioural standards*‘Clinical decision support […] helps not only doctors do things right, but actually to help people do the right thing to their patients, by prompting them [with] rules and so forth in the system. And it was really from there that that the trust became aware that this was a very powerful mover of behaviour within the organization, that actually it may stop mistakes such as patients that are allergic to penicillin getting a penicillin-related compound’. (Consultant physician)	*Professional and relational: seeking to draw on staff’s intrinsic motivation and desire to excel to mobilize improvement*‘We give very local data back to them about their incidents, their complaints, and I expect them to discuss it. So we expect there’s a degree of learning and that seems to work better. […] I think the important thing is actually that it’s not viewed as a corporate responsibility. But actually I like the fact that the wards are competitive against each other and they own the quality in their area, rather than it being a whole-trust thing. ‘Cause otherwise it’s perceived as a corporate behind closed doors and not really applicable to every single frontline worker’. (Clinical director)
Tools deployed	*Communication of standards; surveillance regarding compliance through audits and spot checks*‘There were many people in the organization who didn’t think it would make any difference and badgering everybody about short sleeves and all that, well that’ll just never work, consultants will just not do it. Well, consultants have done it, it’s fine, no problem at all. Well, it has been a problem in some areas, but they’ve been tackled head on as they’ve occurred. […] It’s about a reinforcing and a continual focus on monitoring of that, and so we do that and we do all these spot checks’. (General manager)	*Prescribing system including forcing functions to ‘design out’ error where possible, and permitting the surveillance of prescribing and administration of drugs*‘We’re driving forward quality – and I’m not just saying this ‘cos I’m the informatics bloke – by using data as an evidence base. [Other organizations] are trying to drive forward quality but they are doing case note reviews, or they are looking at paper, or they are relying on incident forms being filled out and reported back to the centre’. (Director of informatics)	*Audits; walkrounds; technologies to make visible and comparable performance on key safety indicators; ‘earned autonomy’*‘[Audits are] monthly if you’re achieving 90% and above. But if you fall below that, they’re fortnightly. If you fall below that they’re weekly. If you fall below that they’re daily. And the ward sisters or department heads are performance managed against that. […] We do a lot of the carrot bit as well. So on all the wards we have these sort of laminated signs of “We’ve now gone however many days without this,” or if the ward has been inspected and found to be [good]. So there’s quite a lot of positive reinforcement of the good behaviour’. (Clinical director)
Resonance of blunt-end frame	*Weak: problems seen as resources and infrastructure; solutions seen as burdensome, distorting and a poor fit for the real challenges*‘We can’t give oxygen unless it’s prescribed; we have got to be assessed on giving oxygen. I have given oxygen for 28 years, and now I am going to get somebody who is probably—I am old enough to be their grandma coming in and seeing if I can put an oxygen mask on somebody correctly. That does hack you off’. (Senior sister)	*Mixed: attention to systems broadly welcomed, but socio-technical approach seen as impacting some groups more than others*‘Poor prescribing is not as obvious on the system, a missed drug is much more obvious, you can pull a report about that and unless you are clinical you are not going to know whether the prescription is poor. It is not as obvious anyway and you certainly don’t spot underlying issues from it and no amount of informatics can pull out every problem’. (Head of quality)	*Strong: importance of patient safety reinforced through lateral influence of colleagues, not just top-down diktat*‘It’s also coming from the side, because they have to work in those departments. And if those departments are saying, “Well, sorry, you’ve got to toe the line, you’ve got to do this,” then you can kick and scream all you like, but you’re just making life uncomfortable for the people around you, and it’s not going to change’. (Risk manager)
Prospects for longevity	*Weak? Reliant on continued active education and enforcement by blunt end*‘Will it settle in time? I hope it will, I hope that people just carry on, because we’ll continue to audit it and we’ll continue to feed back and continue to work on it. Will it be as effective? I don’t know yet. It’s the right way to do it; the real issue is are we trying to do too much too quick?’ (General manager)	*Moderate? Extension of socio-technical solution to wider sharp-end groups challenging*‘In nursing, if someone has not marked off that she has given a drug and they have not even bothered to record, whether it has or hasn’t been given, and then usually that is the night shift, then the following day the other nurses who come on the shift will have to correct that so they can say to those nurses, “I'll have to sort this out for you”, and that works quite well. So your peers actually challenge you on what is acceptable on the ward and in a way we need to get that happening with doctors. I am sure it does happen with some of them but it is how we engage them’. (Head of quality)	*Strong? Some evidence that approach is valued, adopted and owned by the sharp end*‘It’s become so important and embedded that the nurses wanted it [so] that they could capture it electronically rather than manually. So they can trend this up and see what’s happening. So it’s things like that, suddenly you know it’s not—fight isn’t the word. [… But we’ve gone from] “Why do we have to do this?” to “How can we do it better?” And “Actually this needs to be done; we know it needs to be done. How can we do it better? What can we enhance to make it better for us to collect that information? What can we do to the information we’ve got?”’ (Executive director)

### Appleby: the problem of serious incidents and the behavioural solution

At Appleby hospital, a series of serious incidents involving patient harm were assembled into a crisis that triggered the organizational re-centring of patient safety as a problem amenable to organizational intervention. This problematization brought dormant concerns about patient safety to life, raised its profile and created a window of opportunity^7^ in which to convert safety risks into actionable problems.

We just had a little clutch [of incidents] in particular that came together in which – there was almost like an emotional response within the organization, of people saying, ‘Crikey this just isn’t good enough’. (Executive director, Appleby)

The hospital’s senior leadership identified the origin of the problems as primarily lying in deficits in the behaviour of clinical staff. Accordingly, they defined safety as tractable to improvement through changes in rules and their enforcement, which they sought to implement through a managerially-led improvement programme organized around selected themes seen as critical to patient safety, such as oxygen prescribing, improving observations, and identifying and preventing deterioration.

You are responsible for this, and you need to do a, b, c, and d, and we will come back and ask and check, and if you are found to be lacking, then you are responsible. […] It starts with the policy, and then it starts with having people responsible, people understanding what the expectations are of them. (Non-executive Director, Appleby)

The work to improve patient safety was accordingly focused on the *behaviour* of sharp-end staff and the elimination of sub-optimal practices. Individual accountability was strongly emphasized; monitoring of performance, through regular audits and upward accountability, was accompanied by the introduction of reprimands of varying degrees of severity when standards were not met.

I’m very clear that unless you get personal accountability down to an individual level, then I can make all the things around policy change that perhaps have got to improve – [but] unless I’m actually monitoring what individuals are doing, and as we’ve been very clear that this is the standard of practice that we expect, that you just won’t get sustainable, manageable change. (Senior Nurse, Appleby)

Blunt-end participants recognized that a behavioural focus, and accompanying reliance on audit and surveillance for enforcement, might eventually need to be supplemented by efforts to act upon the organization’s culture, but tended still towards an individualizing narrative.

What we’re trying to do is get a message out there which says you’re a part of this organization [which] is taking the patient experience and the quality of patient care very seriously indeed. Success will come from changing that culture so that it becomes important to everybody, rather than endlessly finding more and more things to audit. (Non-executive director, Appleby)

This construction of the problem of safety and its solution by those at the blunt end was not, however, shared uniformly. Interviews with those at the sharp end of clinical practice suggested that they saw the problems as originating not in individuals’ behaviour but in resource constraints, staff shortages and the hospital’s physical infrastructure:

When you visit the ward and you see that there is only one or two members of very stressed looking staff looking after a high case workload, then although I know it’s a good hospital generally, it does generate some concern. (Consultant Physician, Appleby)

For several sharp-end staff, the behavioural focus drew attention away from structural issues in their workplaces. Indeed, some proposed that the organization’s approach aggravated issues they saw as underlying the patient safety problems – for example by adding to workload in a misguided quest for accountability, thereby exacerbating resource problems:

To come and assess somebody and have to go through a three-page assessment on how to give oxygen? I think that is a waste of time and paper. Especially for somebody who has trained as a nurse. (Nurse, Appleby)

More broadly, the improvement programme at Appleby also triggered a degree of ‘initiative fatigue’ among sharp-end staff. In parts of the hospital, clinical staff reported its emphasis seemed misaligned with the goals of their day-to-day work, creating for some a chasm between the concerns of blunt and sharp end.

I think [the focus on the deteriorating patient has] taken away a lot of common sense. […] You talk to management, the people who are bringing this in and going, ‘No, you have to do it then’. But I have to look after my patient first, they are not going to die of a lack of temperature, they are going to die of a lack of oxygen! But some of these people just seem so blinkered. (Nurse, Appleby)

Overall, an unintended consequence of how patient safety was defined as a problem in Appleby, and whose definition got to prevail, was that it risked alienating rather than engaging professional staff, such that ultimately, the effort to revivify patient safety as a focus of attention following the initial crisis was at risk of faltering.

### Berryton: technical opportunities, socio-technical solutions

A different trigger lay behind the problematization process in our second case: Berryton. Here, a new system for electronic prescribing presented the hospital’s blunt-end executives with opportunities to make care safer in two ways: by including ‘forcing functions’ that would prospectively identify contraindications, possible dosing errors and patient allergies; and by developing capacities in the system to identify both individual medication errors and broader patterns relating to drug prescribing and administration.

[The system] was not built to produce those reports. It was built as a clinical system, so Informatics have had to do work on the back end of the system to pull this stuff out because it was not built in that way. (Head of quality, Berryton)

Thus, the IT system produced data that enabled previously occluded problems to be surfaced, such as adverse events, near misses and other safety incidents. Events that reached a threshold of seriousness were treated as crises for the organization that had to be investigated thoroughly and addressed to reduce risk of recurrence. In this sense, the IT system was both the source of, and enlisted in the process of, problematization: it allowed the discovery of hazards and risks not as isolated incidents, but as a way for senior leadership to *continually renew* the problematization of safety. With a problem-generating machine at their disposal, the senior leadership embarked on a sustained programme of root cause analyses (RCAs). The RCAs constructed each incident as a problem to be understood by parsing the contributions of staff behaviour and structural issues (such as poor systems or defective equipment).

If [a failing] is down to you not being bothered, we will take action. If it’s down to you not having the education, we will take action. If it’s down to the system not working properly, we will take action. It’s as much the organization’s fault as the individual. (Executive Director, Berryton)

A second way the system was used to problematize safety was by making visible aspects of routine work where the possibility of intervention through technology now existed. Data from the system allowed insight into compliance with clinical standards (thus qualifying some areas of practice as problems), but at the same time offered the opportunity to ‘design in’^29^ controls and surveillance over the *routine work* of many practitioners in the hospital. This socio-technical construction of the problem of safety meant that, in contrast to Appleby, Berryton sought to transfer some responsibility from clinical staff to IT systems.

The rather different process of problematization, mode of response and implications for the responsibilities of sharp-end staff appeared to result in greater engagement than in Appleby, at least among some groups. More positive attitudes were founded in a sense that the IT systems mitigated some of the challenges of securing safety solely through individuals’ own efforts.

[A nurse] spoke to me about [the IT system]; she feels that it’s good. It ensures safety. She said, ‘It’ll make sure you give [drugs.] It’ll stop you forgetting to give things’. And she liked it. (Fieldnotes, Berryton)

However, here too were challenges. Among these was an increase defensive sharp-end activity in response to the surveillance of the IT system, such as lengthy justifications of actions or non-actions, in response to the risk that their behaviour might be detected as aberrant.

They’re scared, [that’s] why the nurses quite often write an essay […] They write that ‘covering your arse’-type thing’. (Senior Nurse, Berryton)

A second challenge was that the IT system directed attention towards actions and events that were amenable to electronic surveillance. This focused much of the search for solutions on the administration of drugs, and consequently on individuals responsible for these activities. One perverse consequence, found in ethnographic observations and interviews, was that some professional staff (e.g. nurses and pharmacists) were much more exposed than others who might bear equal (or greater) responsibility for patient care.

We started with the nurses, and that’s largely because nurses administer, don’t they, on the whole. I think it’s becoming clear that the doctors have up to now been not been quite so easy to provide the evidence to say, ‘You didn’t do this’. (Senior Nurse, Berryton)

Extending this problematization of safety to those whose work did not fall within the scope of the systems used to enact it was therefore challenging.

### Cherryville: creation of shared mission

The triggers of the problematization of patient safety in Cherryville shared something in common with both Appleby and Berryton. Here, as in Appleby, a series of serious incidents and ‘near misses’ had punctured widely held assumptions that quality and safety were under control, offering a crisis as basis for action. The action itself was led by a newly-appointed chief executive. And, as in Berryton, a new IT system – in this case, for incident reporting – provided the opportunity for continued renewal of knowledge of problems. How Cherryville utilized these windows of opportunity, however, diverged. Here, managers and senior clinicians worked together to use narratives to create shared emotional commitment and sense of mission, and seek input from all quarters on potential solutions. Serious incidents were thus used to both prompt acknowledgement of the existence of a problem *and* facilitate engagement across the staff in developing an appropriate set of solutions.

We launched with an event [where] I talked about a patient who had died of a line-associated septicaemia. ‘Whilst that was happening’, [I said,] ‘we could not say we were a centre of excellence’. So we used that patient’s story. [That] was actually really quite powerful because nobody then could [ask,] ‘Why are you doing this?’ (Executive Director, Cherryville)

At the core of Cherryville’s approach was a recognition of the complexity of the problem of patient safety, and of the limitations of an approach that reduced it to specific issues at the level of behaviour or organizational structure. The participatory process involved a devolution of responsibility for both problems *and solutions* of patient safety to sharp-end staff, supported by a number of interventions that sought to locate ownership of improvement at the sharp end. One, for example, was the introduction of ‘enhanced team leaders’ to oversee resourced improvement projects.

The idea is that each team leader has some jobs that they have to ensure that they do, pretty much continuously. And for almost all the team leaders there are one or two jobs that are specifically around patient safety. (Consultant Surgeon, Cherryville)

Another was the use of the incident reporting system to produce reports on safety used to induce a sense of lateral competition, peer pressure and ownership by showing show staff how well their unit was doing compared with others. Efforts were made to ensure that these initiatives were experienced as exercises in support and mutual understanding, rather than accountability and blame.

The reports now get put on the board about trips and falls every month. And obviously the ward really look to be better than the other wards. Do you know what I mean? So a bit of competition. (Patient liaison lead, Cherryville)

A third effort was greater contact between wards and board, through interventions such as patient safety walk-rounds to demonstrate listening and response to concerns. Senior executives sought to extend a sense of ownership and self-efficacy, in part by collectivizing accountability for the problems of quality and safety and in part by reinforcing the responsibility of clinical staff.

Visiting the area and discussing. Certainly, with my experience working on the ward, the people that work on the ward don't always understand the gravity of things that are happening. They don't understand where that goes, they fill in the incident form and nothing happens. That's what they see. And unless that's brought back to them, by way of visitation or feedback or whatever, they will stop reporting. (Risk manager, Cherryville)

This position appeared to translate into the norms and behaviours of sharp-end staff. There was some scepticism about the motives behind the board’s renewed interest in ward-level activities. However, in contrast to Appleby and Berryton, sharp-end staff in the main appeared to appreciate the approach taken, and value the extent to which they were able to access data, take responsibility for acting on them, and pick up issues with senior managers.

As a clinician and a manager, I can help them pick what those issues are. The support, constant support, but also the pressure, that slight pressure. And I wish I didn’t say it and I wish I didn’t need it, but pressure to keep going. (Clinical nurse specialist, Cherryville)

The trust has been quite good at communicating and having regular briefings about opportunities to even talk to the chief exec, and regular briefings with the execs. So, there’s been quite an open forum of communication. […] There seems to be more openness and two-way discussions, not just told what to do. (Matron, Cherryville)

Cherryville thus sought to ensure that accountability for patient safety resided in the day-to-day professional ethic, vigilance and teamwork of clinical staff, backed up by hands-on attention from the blunt end, so that organizational and clinical goals were aligned.

## Discussion and conclusion

This study suggests that a process of problem definition within organizations may be important in raising the profile of patient safety and to rendering problems amenable to intervention – ‘out of the realm of accident and into the realm of human control’.^12^ Noticing a problem is important, but attention is also needed to *how* problems of patient safety are defined and constructed, because this process influences the selection of strategies for resolving them and the viability of those strategies. Dramatic triggers or crises^28^ may reprioritize safety, but opportunities for improvement may be squandered if they quickly fade in organizational memory, or if problems are constructed in ways that do not achieve the right balance between personal accountability, systems improvement, and use of data and feedback.^30^

To avoid the cycles of problems that emerge into furore and then fade into obscurity, described so well in accounts of the ‘issue-attention cycle’,^8^ purposeful and reflective work is required not just in *recognizing* problems of patient safety but also in *constructing* those problem and their consequences for the solutions and responsibility for realizing them. The precise nature of the crisis is relevant here, but it is not determinative: there are opportunities for intervention by organizational actors to construct the problem in narrower or broader terms. For example, it may be important to avoid framing safety problems as mostly tractable to changes in individual behaviour (as in Appleby), and to manage the prioritization of aspects of care that are easily measured and that risk ‘colonizing’^31^ work time by incentivizing the creation of evidentiary artefacts to head off the blame (as in Berryton).

In this way, our analysis builds on the classic problem definition literature and indicates how its lessons might be applied in understanding the trajectory of problems in health care organizations. Early approaches to understanding social problems^8,32^ presented universal, linear models of the process, whereby issues emerge as problems and then fade away. But the natural history may not be so predictable or consistent: potential problems vie with one another for attention,^28^ and are contested by groups with divergent interests. Important in this regard are the ‘causal stories’ that are inscribed in problems, which ‘assign responsibility for the condition to someone else and so create a burden of reform. People blamed for a problem and saddled with the burden of reform will resist the new causal theory’.^12^ As shown in Table 1, there were commonalities and overlaps in the specific mechanisms used by the three sites. Where they differed, however, was in how they were used to allocate responsibility and ownership of resolution.

Our analysis shows that just as problem definition is a social process, so too is the construction of the underlying causes, the balance of responsibilities, and the most appropriate solution – and critically, these processes offer opportunities for intervention that will affect both the allocation of responsibility and the durability of the problematization. This implies that problem *definition* should be understood as only the initial step in improving quality and safety: the activity that follows, in terms of *constructing* solutions and *distributing* responsibility for implementing them, is also crucial. This implicates not just the ‘pragmatic skills […] of the manager in framing a message’, but also the role of ‘organizational members as active agents’ in the construction of problems and solutions, a feature often neglected in the existing literature.^21^

Our study has important limitations. It is difficult to assess the representativeness of the hospitals studied, though we anticipate that our findings would be transferable. We do not have measures of patient safety across the organizations, and thus cannot determine the extent to which the different approaches impacted on performance. There is a danger that the case-study approach used may lend itself to stories that are too simple, creating the risk of painting the sites as black or white rather than deeply complex. Finally, while we used qualitative data collection techniques in all three sites, the balance between ethnographic observation and interview accounts varied; a different understanding of the construction of problems might have emerged had the balance been different, including a greater sense of the downsides of the approach adopted in Cherryville, where ethnographic observation did not take place.

Useful lessons nonetheless can be drawn from our analysis, adding to the existing literature that highlights the importance of boards’ leadership styles in orchestrating change,^30^ the balance between use of accountability and information systems and reliance on intrinsic motivation,^30,33^ and the engagement of professional groups.^34^ We suggest that those in senior-level roles should not simply identify patient safety as a challenge: they also need to attend carefully to how they frame it as a problem, and how that process influences the choice of therapies. ‘Burning platforms’ – crises that recentre challenging issues and highlight the need for swift action – may be useful devices for those who seek to prioritize quality and safety in health care contexts overwhelmed by ‘priority thickets’.^25^ But these crises may be wasted in the rush to solutions. An approach that promotes shared construction of the problem and solutions, on the other hand, may yield significant benefits. This requires caution and courage, but our data suggest that it may secure a more sustainable re-problematization of quality and safety – rather than creating short-term commotion that quickly becomes part of the noise of competing priorities.
